# Occurrence of *Pseudomonas* spp. in Raw Vegetables: Molecular and Phenotypical Analysis of Their Antimicrobial Resistance and Virulence-Related Traits

**DOI:** 10.3390/ijms222312626

**Published:** 2021-11-23

**Authors:** Lidia Ruiz-Roldán, Beatriz Rojo-Bezares, Carmen Lozano, María López, Gabriela Chichón, Carmen Torres, Yolanda Sáenz

**Affiliations:** 1 Centro de Investigación Biomédica de La Rioja (CIBIR), Área de Microbiología Molecular, C/Piqueras 98, 26006 Logroño, Spain; lidiarroldan@gmail.com (L.R.-R.); brojo@riojasalud.es (B.R.-B.); carmen.lozano@unirioja.es (C.L.); mlopezm@riojasalud.es (M.L.); gchichon@riojasalud.es (G.C.); 2 Área de Bioquímica y Biología Molecular, Universidad de La Rioja, C/Madre de Dios 51, 26006 Logroño, Spain; carmen.torres@unirioja.es

**Keywords:** biofilm, *lasR*, OprD, pigment, ST155, virulence

## Abstract

*Pseudomonas* is characterized by its great capacity to colonize different ecological niches, but also by its antimicrobial resistance and pathogenicity, causing human, animal, or plant diseases. Raw and undercooked food is a potential carrier of foodborne disease. The aim of this study was to determine the occurrence of *Pseudomonas* spp. among raw vegetables, analysing their antimicrobial resistance, virulence, and molecular typing. A total of 163 *Pseudomonas* spp. isolates (12 different species) were recovered from 77 of the 145 analysed samples (53.1%) and were classified into 139 different pulsed-field gel electrophoresis patterns. Low antimicrobial resistance levels, but one multidrug-resistant isolate, were found. Among the 37 recovered *P. aeruginosa* strains, 28 sequence-types and nine serotypes were detected. Eleven OprD patterns and an insertion sequence (IS*Pa1635*) truncating the *oprD* gene of one imipenem-resistant strain were found. Ten virulotypes were observed, including four *exoU*-positive and thirty-one *exoS*-positive strains. The *lasR* gene was absent in three ST155 strains and was truncated by different insertion sequences (IS*Pre2*, IS*1411*, and IS*Pst7*) in other three strains. High biofilm, motility, pigment, elastase, and rhamnolipid production were detected. Our study demonstrated a low occurrence of *P. aeruginosa* (18%) and low antimicrobial resistance, but a high number of virulence-related traits in these *P. aeruginosa* strains, highlighting their pathological importance.

## 1. Introduction

Vegetables and fresh fruit are important products in a healthy diet. In recent years, the search for a good lifestyle has led to an increased consumption of fresh products. Nevertheless, vegetables have become increasingly recognised as potential carriers of foodborne diseases due to various contamination sources, such as dust, soil, manure, irrigation water, or wild animal faeces [1,2]. Moreover, fresh vegetables which are grown close to the soil, are often consumed raw, exposing consumers to the risk of infection [3]. Most foodborne diseases are not reported, and sometimes outbreaks may affect a wide number of people. Thus, there is a special interest to know the epidemiology and spread of foodborne pathogens that are adaptable to different environmental conditions [4]. Additionally, bacteria can develop antimicrobial resistance due to spontaneous mutations or acquisition of resistance mechanisms by horizontal gene transfer. Antimicrobial resistant isolates can also spread antimicrobial resistance genes to other commensal and pathogenic bacteria [5]. The role of food in human exposure to antimicrobial resistant bacteria, as well as a reservoir of resistance genes, is becoming a growing food safety issue [3].

*Pseudomonas* is a non-fermenting Gram-negative bacterium that colonizes different niches, due to their metabolic capacity and broad potential for adaptation to different conditions [6]. This genus includes a wide variety of species, *Pseudomonas aeruginosa* being the most important one. This species is a major opportunistic human pathogen with increasing medical and veterinary importance. The significance of *P. aeruginosa* is marked by its great resistance to antimicrobials and antiseptics and the presence of multiple virulence factors [6]. *P. aeruginosa* uses its big arsenal of pathogenicity factors (including adhesins and secretion toxins, effector proteins, proteases, elastases and pigments) to interfere with host defences. The type 3 secretion system (T3SS) is a major virulence weapon that contributes to cytotoxicity and acute infections, injecting potent exotoxins called effectors (ExoU, ExoS, ExoY and ExoT) into cytoplasm of the host cell due to its syringe form [6,7]. The ExoU effector is associated with an increased risk of early clinical mortality [8,9]. Furthermore, it has been demonstrated that the predominance and persistence of this species in food and on surfaces of food processing plants is related to its ability to form biofilm. Most of these virulence factors are under the control of a cell density recognition mechanism called Quorum-Sensing (QS) [10,11]. *P. aeruginosa* possesses two well-defined and interrelated QS systems, *las* and *rhl*, which are used to regulate gene expression through the production and secretion of autoinducers, by *lasI* and *rhlI* genes, activating LasR and RhlR regulators, respectively [10].

Previous studies have given information about the detection of *Pseudomonas* spp. in food of animal or vegetable origin [1,3,12,13], but there are little data about their antimicrobial resistance and virulence phenotype. For this reason, the purpose of this study was to analyse and characterise the *Pseudomonas* spp. population in food vegetables, at both genotypic and phenotypic levels.

## 2. Results

### 2.1. Isolates of Pseudomonas spp.

In total, 163 *Pseudomonas* spp. were isolated from 77 vegetal samples (prevalence of 53.1%), belonging to (number of positive samples): lettuce (18), chard (14), potato (11), green bean (10), cucumber (9), zucchini (8) and onion/leek (7) (Table S1). Regarding each sample type, the highest prevalence was detected in lettuce (90%) and chard (70%) samples, and the lowest one was among the onion/leek samples (26.3%).

The 163 isolates were classified into twelve different *Pseudomonas* species. *P. putida* (51 isolates), *P. aeruginosa* (50 isolates) and *P. mendocina* (32 isolates) were the most abundant ones and were isolated from 46, 26 and 29 samples, respectively (Table 1). Thirty samples harboured more than one different *Pseudomonas* species (Table S1).

### 2.2. Antimicrobial Susceptibility Testing

The 163 *Pseudomonas* spp. isolates showed the following resistance percentages to: aztreonam (42%), imipenem (1.8%), meropenem (1.8%), doripenem (1.8%), piperacillin (0.6%), and ceftazidime (0.6%). The isolates showed susceptibility to the remaining seven antibiotics tested. Fifty-four per cent of the isolates were susceptible to all antibiotics tested (Table S1). Conversely, one *P. fluorescens* isolate (Ps876), recovered from a zucchini collected in an orchard, showed a multidrug-resistance phenotype, showing resistance to imipenem, meropenem, doripenem, ceftazidime and aztreonam (Table S1).

None of the 163 isolates showed class A carbapenemase, metallo-beta-lactamase (MBL) or extended spectrum beta-lactamase (ESBL) phenotypes, whereas the AmpC inducible phenotype was detected in 31% of the isolates, which included all *P. aeruginosa* isolates and the multidrug resistant *P. fluorescens* isolate (Ps876) (Table S1).

### 2.3. Molecular Typing

One hundred and thirty-nine different pulsed-field gel electrophoresis (PFGE) patterns were detected among the 163 *Pseudomonas* spp. (Table S1), and regarding the 50 *P. aeruginosa* isolates, 36 PFGE patterns were observed (Table 2). Indistinguishable patterns were only detected among isolates from the same sample, selecting one strain for next steps; except for two *P. aeruginosa* isolates that showed the same PFGE pattern, but they were recovered from two different chard samples (Table 2 and Table S1). One strain with different PFGE pattern and species per sample were included in further studies. Additionally, three *P. putida* isolates from the same cucumber were included because they showed the same PFGE profiles but different resistance phenotypes. After these criteria, 142 *Pseudomonas* spp. strains were chosen: 105 strains corresponding to *Pseudomonas* non-*aeruginosa* species and 37 *P. aeruginosa* strains (Table S1).

**Figure 1:**
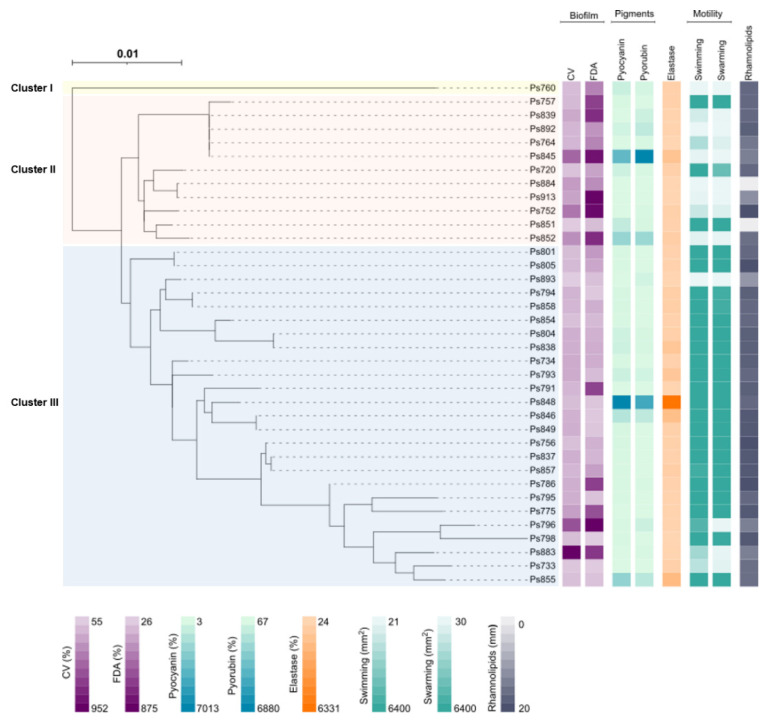
Maximum-likelihood phylogenetic tree based on the sequence type (MLST) and phenotypic characteristics of the 37 *P. aeruginosa* strains analysed in this study. The phylogenetic tree was obtained using the IQTREE v.1.6.1 [14] software. The iTol v.4 [15] program was used to visualise the phylogenetic tree and to perform the eight heatmaps. In order: biofilm biomass production (CV), bacterial metabolic activity inside the biofilm (FDA), pyocyanin and pyorubin production, elastase activity, swimming and swarming motility and rhamnolipids production. Legend values belong to the minimum and maximum data for each phenotypic assay (Table 2). The three clusters (I, II and III) were marked with different colours: yellow (Cluster I), red (Cluster II) and blue (Cluster III).

Twenty-eight different sequence types (ST) were determined among the 37 *P. aeruginosa* strains using Multilocus sequence typing (MLST) method (Table 2). Eight of them (ST2416, ST2427-ST2432 and ST2448) were first described in this study and named by the MLST database. Seven ST were repeated more than once: ST155, ST274, ST982, ST1226, ST1228, ST2416 and ST2432 (Table 2 and Figure 1). The *P. aeruginosa* strains were distributed into three clusters (Cluster I, II and III) when a phylogenetic tree based on the MLST was obtained (Figure 1). Cluster I was an outlier and only included the Ps760 strain (ST2448). Cluster II included the new high-risk clone ST155, whereas the cluster III possessed the intercontinental clones, ST253, ST274 and ST395. The new ST were distributed between both clusters.

### 2.4. Serotyping

Nine different serotypes were identified in these 37 *P. aeruginosa* strains, in addition to five non-agglutinable (Ps798, Ps839, Ps884, Ps892 and Ps913) and one poly-agglutinable (Ps796) strains. Serotype O:6 was the most predominant (40.5%), followed by O:5 (10.8%) and O:1 (8.1%) (Table 2). Serotypes O:2, O:7, O:10, O:12; O:13, O:14 and O:15 were not found in this study. Strains belonging to the same ST showed the same serotype, except for ST155 strains that were non-agglutinable or O:6 serotype (Table 2).

### 2.5. Characterisation of Porin OprD

The *oprD* gene was amplified in all *P. aeruginosa* strains, and eleven amino acidic OprD profiles were distinguished (Table 2 and Table S2). Only two strains had the same pattern as *P. aeruginosa* PAO1 (pattern A, wild type), and pattern B was the most frequently detected (23 strains). The deletion of two amino acids in the region from amino acid 372 to 383 of the loop 7 (loop L7-short), which encodes a protein OprD of 441 amino acids, was identified in 24 strains (64.8%) (patterns B and E) (Table 2 and Table S2). Regarding the two imipenem-resistant strains, pattern B was observed in Ps839 strain, and the Ps884 strain showed the *oprD* gene truncated by the insertion sequence IS*Pa1635* at nucleotide position 561 (pattern K). This insertion sequence belongs to the IS*4* family, and this is the first description of IS*Pa1635* truncating *oprD* gene. Thus, the sequence was included in GenBank with the accession number MH050332.

A total of 11 *P. aeruginosa* strains (one per pattern) with different protein OprD profiles were selected to study their outer membrane proteins by SDS-PAGE. The OprD band was detected in all tested strains except in pattern K, which corresponded to the strain with IS*Pa1635* element truncating the *oprD* gene.

### 2.6. Virulence Patterns

**Figure 2:**
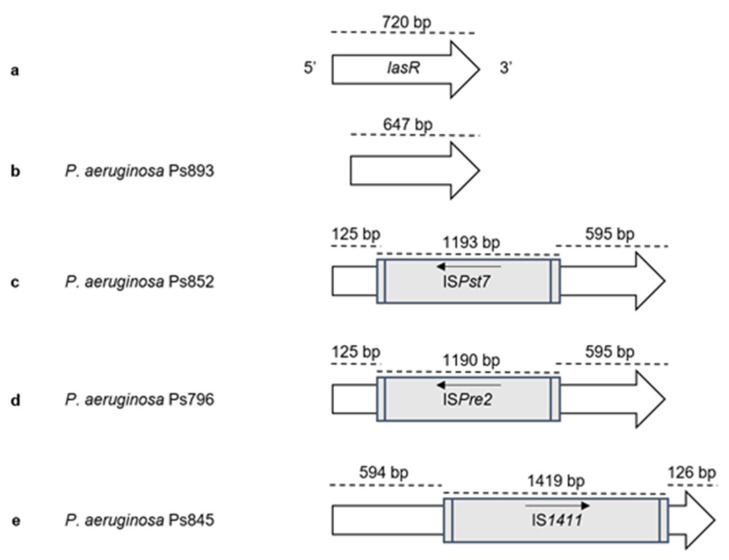
Representation of truncated *lasR* genes found among our *P. aeruginosa* strains. The position and orientation of the gene and insertion sequences are indicated by arrows: (**a**) *lasR* gene (PA1430) of the control strain *P. aeruginosa* PAO1 (NCBI accession number NC_002516); (**b**) Ps893 strain, *lasR* size is 647 bp due to the presence of a deletion at the beginning of the gene; (**c**) Ps852 strain, *lasR* truncated at position 125 bp by the insertion sequence IS*Pst7* (1193 bp) (GenBank accession number MH050331); (**d**) Ps796 strain, *lasR* truncated at position 125 bp by the insertion sequence IS*Pre2* (1190 bp) (GenBank accession number MH050329); (**e**) Ps845 strain, *lasR* truncated at position 595 bp by the insertion sequence IS*1411* (1419 bp) (GenBank accession number MH050330).

The presence of virulence and QS genes was investigated in the 37 *P. aeruginosa* strains, and ten different virulence patterns were obtained (Table 2 and Table S3). Regarding the T3SS, *exoU* gene was detected in 4 *P. aeruginosa* strains and *exoS* in 31 strains. Neither *exoU* nor *exoS* genes were amplified in two strains (pattern VII). Moreover, *exoA* and *exoY* genes did not amplify in one and three strains, respectively. The *exoT*, *lasA*, *lasB*, *aprA, rhlAB, rhlC, rhlI*, and *rhlR* genes were detected in all strains, whereas *exlA* gene was absent in all of them. The *lasI* and *lasR* genes, involved in the QS system, were not amplified in three strains (pattern IV) that belonged to ST155 (Ps764, Ps839, Ps892). In the remaining ST155 strain (Ps845), as well as in two more strains (Ps796, Ps852), the *lasR* amplicon sized higher than 2,000 bp, resulting in the first description of three insertion sequences (IS*1411,* IS*Pre2*, and IS*Pst7*, respectively) and truncating this gene (Table 2 and Figure 2). All *lasR* sequences were submitted in GenBank (accession number): IS*1411* (MH050330), IS*Pre2* (MH050329), and IS*Pst7* (MH050331). Finally, the *lasR* gene of Ps893 strain sized lower than expected, 647 instead of 720 bp, due to the presence of a deletion at the beginning of the gene (Figure 2).

### 2.7. Biofilm Quantification

Table 2 and Figure 1 summarise the biofilm biomass production (CV) and the bacterial metabolic activity inside the biofilm (FDA) of the 37 *P. aeruginosa* strains. The 92% of *P. aeruginosa* strains displayed higher values for biomass biofilm production than *P. aeruginosa* PAO1. Ps883 and Ps796 strains showed the highest percentages (952% and 600%, respectively), whereas Ps893, Ps851 and Ps733 exhibited the lowest ones (56, 65 and 90%, respectively). For FDA assay, 73% of strains showed more metabolic activity than the control strain *P. aeruginosa* PAO1. Ps752, Ps796, Ps845, Ps852 and Ps913 were the highest producers (>700%), and Ps798 the lowest producer (26.2%). Likewise, strains that had an absent or truncated *lasR* gene showed high levels of biomass production and bacterial metabolic activity inside the biofilm, except for Ps893 that showed low levels in comparison with reference *P. aeruginosa* PAO1 strain.

### 2.8. Motility

The different swarming and swimming patterns detected among the 37 *P. aeruginosa* strains are included in Figure S1. Analysing the swimming results, many strains belonging to the Cluster III showed higher motility than the remaining ones (Table 2 and Figure 1). In fact, 23 of 37 strains (62.1%) covered the entire Petri dish surface (5363.7 to 6400 mm^2^) in swimming and swarming. Ps893 and Ps884 strains described the lowest swimming (21.6 and 25.2 mm^2^) and swarming (30.0 and 38.3 mm^2^) values (Table 2 and Figure 1). Ps796 showed a high value of swimming (5363.7 mm^2^) but showed a low swarming value (97.2 mm^2^).

*P. aeruginosa* strains having an absent or truncated *lasR* gene showed medium and low levels of motility, except the Ps796 swimming (Table 2 and Figure 1). Ps892 (absent gene) and Ps893 (truncated gene) showed the lowest motility values. Considering the strains which carried an insertion sequence truncating the *lasR* gene (patterns IIa, IIb and VI), the swarming levels were lower than the swimming motility ones.

### 2.9. Elastase and Pigment Production

Results for quantification of pyorubin and pyocyanin production as well as elastase activity are summarised in Table 2 and Figure 1.

For pyorubin assay, 70.2% of strains showed high levels of production in comparison with *P. aeruginosa* PAO1. Besides, Ps845, Ps848 and Ps852 showed the highest levels of pyorubin. Conversely, 27% of strains were the lowest pyorubin producers, highlighting Ps733, Ps775 and Ps854 strains. For the pyocyanin values, 62% showed high levels of production, being Ps845 and Ps848 the most producers. Conversely, fourteen strains showed low levels in comparison with reference strain, and Ps883, Ps884 and Ps913 strains were the weakest producers. The four strains with an absent or truncated *lasR* gene showed low levels of pyocyanin production.

Regarding elastase activity assay, 83.7% of strains showed high levels of activity in comparison with *P. aeruginosa* PAO1. Ps846 and Ps855 were the most important elastase producers (Table 2 and Figure 1).

### 2.10. Rhamnolipids Detection

Figure S2 shows some of the results obtained with the rhamnolipid assays in *P. aeruginosa* strains. The 94.6% of the strains showed halos ≥11 mm (the minimum value observed), many of them (10 strains) with halos ranging 19–20 mm of diameter (Table 2 and Figure 1). Ps851 and Ps884 did not produce rhamnolipids.

## 3. Discussion

Food and the environment have been described as reservoirs of bacteria harbouring antimicrobial resistance genes that could be transferred or mobilised into human pathogens. Moreover, the extensive use and even the misuse of antimicrobial agents in clinic, animal production, and agriculture could be a way to select and disseminate these resistant human pathogens [5], and among them *Pseudomonas* genus. Some reports showed different clinical cases caused by environmental *Pseudomonas*, such as *P. mendocina*, *P. monteilii* or *P. putida* [16,17,18,19], although *P. aeruginosa* is the most important pathogenic bacterium. In our study, the 53.1% of fresh vegetables were positive for *Pseudomonas* spp., and lettuce and chard were the most frequently contaminated vegetables, as well as in previous studies [12,13]. According to our results and previous reports [1,20,21], the vegetables cultivated in contact with the soil may be contaminated more easily with *Pseudomonas* coming from soil, fertilizers, manure or water used for irrigation. In contrast, the lowest presence of *Pseudomonas* spp. was among onion samples. This fact could be due to the layered structure of the onion and/or the bioactive compounds present in onions [22,23,24]. Previous studies revealed that fresh onions, even onion wastes, exhibited high antimicrobial activity against bacteria such as *Escherichia coli*, *P. fluorescens* and *Bacillus cereus*, among others.

Antibiotic susceptibility testing revealed that all *Pseudomonas* spp. were susceptible to aminoglycosides and fluoroquinolones, and they showed low resistance rates for aztreonam and carbapenems. Only two imipenem-resistant *P. aeruginosa* strains were detected, and none of them were an MBL producer. The imipenem-resistance of Ps884 strain was associated with the loss of function of its OprD porin due to the truncation of the *oprD* gene by the insertion sequence IS*Pa1635*. The inactivation of this porin gene by insertion sequences has been deeply studied in clinical strains [25,26,27], but this is the first time that the IS*Pa1635* has been identified in a *P. aeruginosa* from food origin and truncating *oprD* gene. Conversely, the imipenem-resistant Ps839 strain showed the same amino acid changes detected in the OprD porin as those reported in carbapenem-susceptible *P. aeruginosa* isolates [28,29]. Thus, other resistance mechanisms such as active efflux pumps or AmpC hyperproduction could be involved in that phenotype.

Considering *P. aeruginosa* QS genes, *rhlI* and *rhlR* genes were amplified in all *P. aeruginosa* strains. However, *lasI* and *lasR* genes were not detected in three strains; the other three strains showed insertion sequences truncating the *lasR* gene (IS*1411*, IS*Pst7* and IS*Pre2*), and one strain showed a short *lasR* gene leading the possibility of losing the QS function. Several reports have mentioned the frequency of *lasI* and *lasR* mutations, or the lack of *lasR* gene among clinical and environmental isolates to favour their adaptation or persistence [30,31,32]. They have also demonstrated that a mutation in *lasR* does not lead to virulence factors loss, due to the regulation mediated by the *rhl* system, taking control of the phenazines production or rhamnolipids synthesis [10]. In addition, there are two other QS mechanisms, the *Pseudomonas* quinolone signal (PQS) and the integrated quorum sensing (IQS) mechanism, able to replace, in many cases, the LasR function [6,10]. Regarding our phenotypic results, it is important to remark that a high percentage of analysed *P. aeruginosa* showed high levels of biofilm, pigments and rhamnolipids production, and elastase activity even those with an absent or truncated *lasR* gene; however, in these cases, the strains were not as mobile as the remaining strains. Nevertheless, the hypothesis of the action of other QS mechanisms could demonstrate the pathological importance of these *P. aeruginosa* strains.

In *P. aeruginosa*, the T3SS mechanism contributes to cytotoxicity and acute infections [6,7]. The *exoU* gene was detected in four strains, all of them situated in the same branch of the MLST cluster, including the ST253 [33] and the new one ST2427, and belonging to O:11 serotype or poly-agglutinable, as other reports [34]. Usually, the *exoU* gene is described in clinical isolates, but it has also been detected in environmental strains [29,35].

The pathogenicity and host adaptation of *P. aeruginosa* is associated with its worldwide dissemination and specific sequence types. Among all sequence types detected, unlike other reports regarding clinical isolates, the most important “high-risk clones” ST111, ST175 and ST235, were not found. However, intercontinental clones disseminated worldwide, newest high-risk clones, such as ST155 and ST244 [36,37], as well as the epidemic clone ST274 circulating in Spain [38,39], were detected. All of those, including ST252, ST253, and ST395 epidemic clones were also previously observed in clinical animal and environmental samples [36,40,41,42]. However, none of the epidemic clones showed the same pathogen phenotype. In this case, the low motility but high biofilm production that possessed the STs from the second cluster, where ST155 clone was included. Conversely, the *P. aeruginosa* strains belonging to the third cluster exhibited higher motility, where high-risk clones ST253, ST274 and ST395 were englobed. However, there are some exceptions, such as *P. aeruginosa* strains with lacked or truncated *lasR* gene, Ps852 (ST267), Ps893 (ST395) or Ps764, Ps839, Ps845 and Ps892 (ST155). Further studies are needed to delve into the relationship between the genotype and phenotype of these epidemic clones because, to the best of our knowledge, this is the first time the analysis of this relationship is described in environmental samples.

## 4. Materials and Methods

### 4.1. Bacterial Isolates

One hundred and forty-five samples of raw vegetables were recovered from orchards (82 samples) and little markets (63 samples) of different areas of La Rioja region (Spain), during 2015. Samples were divided as follows: 20 lettuces (*Lactuca sativa*), 22 cucumbers (*Cucumis sativus*), 20 zucchinis (*Cucurbita pepo*), 23 onions/leeks (*Allium cepa*/*Allium ampeloprasum* var. *porrum*), 20 potatoes (*Solanum tuberosum*), 20 green beans (*Phaseolus vulgaris*) and 20 chards (*Beta vulgaris* var. *cicla*).

A quantity of 30–35 g of each sample was enriched in 100 mL of Tryptose Soy Broth (Becton Dickinson, Franklin Lake, NJ, USA), and homogenised in a stomacher. A suspension volume (40 mL) was incubated at 37 °C during 24 h in agitation. Then, 100 µL of this suspension was streaked onto Cetrimide-agar plates (Becton Dickinson, Le Pont de Claix, France) and incubated at 42 °C during 24–48 h. Two or three different colonies per plate, presumptive of being *Pseudomonas*, were selected, identified by classical biochemical methods (Triple Sugar Iron and oxidase tests), and confirmed by PCR amplification and sequencing of 16S rRNA fragment [28] and by Matrix-Assisted Laser Desorption/Ionization Time-of-Flight (MALDI-TOF) mass spectrometry (Bruker, Billerica, MA, USA).

### 4.2. Antimicrobial Susceptibility Testing

Antimicrobial susceptibility testing was performed by disc diffusion method following the Clinical and Laboratory Standards Institute guidelines [43]. Thirteen antipseudomonal agents were tested (disc concentration), including piperacillin-tazobactam (100/10 µg), piperacillin (100 µg), aztreonam (30 µg), cefepime (30 µg), ceftazidime (30 µg), imipenem (10 µg), meropenem (10 µg), doripenem (10 µg), tobramycin (10 µg), gentamicin (10 µg), amikacin (30 µg), netilmicin (30 µg), and ciprofloxacin (5 µg). ESBL, MBL, class A carbapenemase and inducible AmpC phenotypes were determined by double-disc synergy tests [29].

### 4.3. Molecular Typing

The clonal relationship among the recovered isolates was determined by PFGE with *SpeI* restriction enzyme [28]. PFGE patterns were analysed by the Java program GelJ using the Dice coefficient [44].

MLST for *P. aeruginosa* was performed by PCR and sequencing [29,45]. Allelic profiles and sequence types (STs) were assigned according to the PubMLST database (http://pubmlst.org/paeruginosa/ accessed on April 2016). A maximum-likelihood phylogenetic tree, relating the sequence types of the *P. aeruginosa* strains, was performed using IQTREE v.1.6.1 [14], and visualised with iTol v.4 [15].

### 4.4. Serotyping

*P. aeruginosa* strains were serotyped by slide agglutination according to the International Antigenic Typing Scheme (IATS), using 16 type O monovalent antisera specific for *P. aeruginosa* (O:1 to O:16) following the manufacturer’s protocol (Bio-Rad, Temse, Belgium).

### 4.5. Characterisation of Porin OprD

Amino acid changes of the porin OprD were analysed by PCR and sequencing in all *P. aeruginosa* strains [29]. The mutations were determined by comparison with the sequence of the control strain *P. aeruginosa* PAO1 (GenBank accession number AE004091).

The outer membrane proteins (OMPs) of selected strains were stained with Coomassie Brilliant Blue and were visualised by SDS-PAGE (Sodium Dodecyl Sulphate-PolyAcrylamide Gel Electrophoresis) in a Bio-Rad Mini-Protean II apparatus (Bio-Rad, Temse, Belgium) as previously described [46]. *P. aeruginosa* PAO1 and PAO1 lacking-OprD were included as control strains.

### 4.6. Detection of Virulence Marker Genes

The presence of *exoU, exoS, exoY, exoT, exlA, exoA, lasA, lasB, aprA, rhlAB, rhlC, rhlI, rhlR, lasI*, and *lasR* virulence and QS genes was analysed by PCR in *P. aeruginosa* strains [47].

### 4.7. Biofilm Quantification

The analysis of the total biofilm biomass was performed by crystal violet (CV) staining, and the bacterial metabolic activity inside the biofilm structure by fluorescein diacetate (FDA) assay among *P. aeruginosa* strains [47]. Both methods were carried out in flat-bottom microtiter 96-well plates after 24 h of bacterial incubation in Müeller–Hinton broth at 37 °C. For CV assay, 66% acetic acid and 10% CV were used, and the FDA working solution concentration was 0.1 mg/mL. Measures were performed using a POLARstar Omega microplate reader (BMG Labtech, Ortenberg, Germany). All assays were carried out in triplicate, including *P. aeruginosa* PAO1 as control.

### 4.8. Motility

Swarming and swimming motilities were studied in *P. aeruginosa* strains [47], placing 4 µL of bacterial suspension (1 × 10^9^ cells in Luria–Bertani (LB) broth) on the middle of 0.5% (swarming) and 0.3% (swimming) LB agar plates and subsequent incubation at 37 °C overnight. The plates were imaged with Chemi Doc system (Bio-Rad, Temse, Belgium), and processed with Image Lab software (version 6.0.1, Bio-Rad). The entire plate area was 6400 mm^2^. All assays were performed in triplicate, including *P. aeruginosa* PAO1 as a control strain.

### 4.9. Elastase and Pigment Production

Bacteria were grown overnight in LB broth at 37 °C with shaking at 120 rpm. After centrifugation, 900 μL of supernatant was used to determine the elastase activity in *P. aeruginosa* strains by the Elastin Congo Red assay as previously described [48].

The chloroform extract method was used to quantify pyocyanin and pyorubin phenazines by measuring the absorbance of the corresponding solutions: the organic phase at 520 for pyocyanin, and the aqueous phase at 525 nm for pyorubin, using a POLARstar Omega microplate reader (BMG Labtech, Ortenberg, Germany) [49], and including *P. aeruginosa* PAO1 as control.

### 4.10. Rhamnolipids Detection

The detection of *P. aeruginosa* biosurfactant producers was carried out by the Cetyl Trimethylammonium Bromide–Methylene Blue (CTAB-MB) agar plates method [50,51,52]. One colony of each *P. aeruginosa* strain studied was inoculated in 3 mL of mineral salt medium (MSM) broth and was incubated at 35 °C and 130 rpm during 48 h. Following previous procedures, shallow wells of 6 mm were cut on the CTAB-MB agar plate surface. Twenty microliters of the inoculum were added into each well. The plates were incubated for 24–48 h at 35 °C, and then stored in the fridge for at least 24 h, to intensify the blue colour of the plates to facilitate the recognition of the rhamnolipids production. The halo was measured (mm). *P. aeruginosa* PAO1 was used as a positive control.

## 5. Conclusions

*Pseudomonas* strains contaminating fresh vegetables were found in this work, especially in lettuce and chard. A variety of different *Pseudomonas* species, including pathogenic to humans, such as *P. aeruginosa, P. mendocina*, *P. monteilii* or *P. putida* were detected. *P. aeruginosa* was recovered from 26 (17.9 %) of the vegetable samples and belonged to many different clones, comprising some international clones. Moreover, these strains showed low resistance to antibiotics but high presence of virulence-related traits, as high biofilm, pigments and rhamnolipids production. *P. aeruginosa* is an opportunistic human pathogen, and the food chain might be a source of transmission to humans. Identifying the natural reservoirs of this important pathogen and elucidating its molecular biology are crucial tasks in the pursuit of minimising its transmission. For all these reasons, the application of proper hygiene practices along the food production/supply chain is essential, not only for vegetable workers, but also for consumers.

## Figures and Tables

**Table 1:** Description of the twelve different *Pseudomonas* species isolated from raw vegetables, including the number of isolates and the samples where they were recovered.

Species	No. Isolates	Samples (No.)
*P. putida*	51	Chard (11), potato (8), zucchini (7), cucumber (6), lettuce (5), green bean (5), onion/leek (4)
*P. aeruginosa*	50	Chard (7), lettuce (7), green bean (5), potato (2), zucchini (2), cucumber (2), onion (1)
*P. mendocina*	32	Lettuce (16), chard (12), potato (1)
*P. plecoglossicida*	17	Lettuce (5), cucumber (4), chard (3), potato (3), onion/leek (1), green bean (1)
*P. monteilii*	6	Zucchini (2), onion/leek (2), cucumber (1), chard (1)
*P. alcaligenes*	1	Lettuce (1)
*P. alcaliphila*	1	Cucumber (1)
*P. chlororaphis*	1	Potato (1)
*P. fluorescens*	1	Zucchini (1)
*P. oryzihabitans*	1	Green bean (1)
*P. otitidis*	1	Lettuce (1)
*P. punonensis*	1	Green bean (1)
	**163**	

**Table 2:** Genotypic and phenotypic characteristics of the 37 *P. aeruginosa* strains recovered from food vegetables.

Strain	Origin	PFGE	Serotype ^a^	MLST	Resistance Phenotype ^a^	OprD Pattern ^b^	Virulence Pattern ^c^	Biofilm (%) ^d^		Pigments (%) ^d^		Elastase (%) ^d^	Motility (mm^2^)		Rhamnolipids (mm)
								CV	FDA	Pyocyanin	Pyorubin		Swimming	Swarming	
**Ps720**	Cucumber	P11	O:5	ST277	-	F	I	103.1	197.2	443.4	154.7	390.0	6400.0	4637.6	17
**Ps733**	Lettuce	P1	O:11	ST2427 ^e^	-	D	VIII	90.0	42.4	68.6	67.6	376.2	1748.6	383.9	16
**Ps734**	Lettuce	P12	O:6	ST1090	-	B	I	204.0	161.4	91.0	71.7	240.2	6400.0	6400.0	18
**Ps752**	Chard	P2	O:6	ST385	-	B	I	434.3	846.8	51.4	138.2	326.9	1267.5	555.4	20
**Ps756**	Potato	P3	O:6	ST2429 ^e^	-	B	I	113.0	142.3	144.1	93.4	279.8	6400.0	6400.0	20
**Ps757**	Potato	P5	O:6	ST2430 ^e^	-	B	I	138.8	609.8	101.7	84.3	368.6	6400.0	6400.0	17
**Ps760**	Lettuce	P24	O:8	ST2448 ^e^	-	H	III	136.4	333.5	607.9	296.2	380.6	68.7	40.1	17
**Ps764**	Onion	P25	O:6	ST155	-	B	IV	157.0	325.0	22.8	167.0	51.9	2158.5	542.8	15
**Ps775**	Lettuce	P4	O:6	ST782	-	B	I	260.6	547.3	62.3	68.5	158.5	6400.0	6400.0	19
**Ps786**	Lettuce	P26	O:9	ST2351	-	B	I	190.0	620.4	127.7	142.6	228.9	6400.0	6400.0	20
**Ps791**	Potato	P6	O:4	ST1033	-	B	I	155.2	603.4	45.0	111.5	133.5	6400.0	6400.0	18
**Ps793**	Potato	P7	O:5	ST2428 ^e^	-	E	I	203.0	101.1	557.4	347.3	948.7	6400.0	6400.0	17
**Ps794**	Lettuce	P8	O:3	ST274	-	B	I	167.1	43.2	149.5	106.5	420.9	6400.0	5969.0	18
**Ps795**	Lettuce	P9	O:6	ST1135	-	B	I	190.0	72.7	84.1	100.0	170.8	6400.0	6400.0	17
**Ps796**	Green bean	P27	PA	ST2411	MEM; ATM	C	VI	600.0	875.7	91.8	600.5	167.6	5363.7	97.2	14
**Ps798**	Green bean	P28	NA	ST2431	-	B	III	121.3	26.2	123.2	135.2	74.4	6400.0	6267.7	19
**Ps801**	Green bean	P29	O:6	ST2432 ^e^	-	B	I	123.8	243.8	141.5	134.2	246.7	6400.0	6400.0	17
**Ps804**	Cucumber	P30	O:6	ST982	-	B	I	189.4	138.0	355.7	213.2	350.3	6400.0	6400.0	18
**Ps805**	Lettuce	P10	O:6	ST2432 ^e^	-	B	I	141.7	183.6	92.7	95.2	214.3	6400.0	6400.0	20
**Ps837**	Green bean	P15	O:6	ST1226	-	B	I	184.4	125.8	120.1	76.9	260.9	6370.2	6400.0	19
**Ps838**	Chard	P13	O:6	ST982	-	B	I	188.8	155.2	481.3	266.6	1017.3	6400.0	6400.0	18
**Ps839**	Chard	P14	NA	ST155	IPM	B	IV	208.5	694.0	66.9	606.8	162.2	865.7	134.3	17
**Ps845**	Chard	P16	O:6	ST155	-	B	IIa	510.6	801.9	3877.5	6880.3	1195.7	272.4	56.1	14
**Ps846**	Chard	P22 *	O:1	ST1228	-	I	I	174.8	46.4	1249.9	980.1	1535.7	6400.0	6400.0	19
**Ps848**	Zucchini	P17	O:11	ST1232	-	G	I	126.3	54.4	7013.9	4902.2	6331.2	6400.0	6400.0	17
**Ps849**	Chard	P22 *	O:1	ST1228	-	I	I	186.7	52.3	154.8	121.5	281.6	6400.0	6400.0	19
**Ps851**	Green bean	P18	O:5	ST244	-	A	I	65.0	89.9	780.9	229.6	265.7	6400.0	6400.0	-
**Ps852**	Green bean	P19	O:5	ST267	-	J	IIb	332.4	700.4	2245.2	2070.5	493.9	342.1	167.5	16
**Ps854**	Zucchini	P20	O:1	ST252	-	B	I	118.1	103.7	132.3	69.9	231.7	6400.0	6400.0	17
**Ps855**	Zucchini	P21	O:16	ST253	-	C	V	112.8	75.2	2355.4	1119.6	1750.2	6400.0	6400.0	16
**Ps857**	Chard	P23	O:6	ST1226	-	B	I	157.6	219.7	111.5	81.5	482.9	6014.5	6400.0	19
**Ps858**	Chard	P31	O:3	ST274	-	B	I	159.0	148.6	166.5	111.1	430.4	6400.0	5892.1	17
**Ps883**	Zucchini	P32	O:11	ST1284	-	C	V	952.1	652.8	7.6	144.7	95.6	2532.6	187.6	14
**Ps884**	Green bean	P33	NA	ST2416 ^e^	IPM	K	VII	276.4	292.4	4.8	133.5	25.0	25.2	38.3	-
**Ps892**	Chard	P34	NA	ST155	-	B	IV	155.5	259.1	335.2	1005.7	210.8	84.5	69.8	18
**Ps893**	Chard	P35	O:6	ST395	-	B	IIc	55.8	85.3	13.2	382.2	55.9	21.6	30.0	11
**Ps913**	Chard	P36	NA	ST2416 ^e^	-	A	VII	241.3	863.8	3.8	88.1	24.5	261.6	48.4	12

^a^ NA, non-agglutinable; PA, poly-agglutinable; -, this strain was susceptible to all 13 antibiotics tested; ATM, aztreonam; IPM, imipenem; MEM, meropenem. ^b^ The OprD patterns are described in Table S2. ^c^ Virulence profiles are defined in Table S3. ^d^ Percentages determined by comparison with *P. aeruginosa* PAO1 (100%). ^e^ New MLST. * Ps846 and Ps849 showed the same PFGE pattern, but they were recovered from different chard samples (Table S1).

## Data Availability

All datasets are available. New sequences of *lasR* genes truncated by insertion sequence elements were submitted in GenBank (accession number): IS*1411* (MH050330), IS*Pre2* (MH050329), and IS*Pst7* (MH050331). The *oprD* gene truncated by the insertion sequence IS*Pa1635* was included in GenBank with the accession number MH050332.

## Supplementary Materials

The following are available online at https://www.mdpi.com/article/10.3390/ijms222312626/s1.
