# Supplementary material for: Occurrence of Pseudomonas spp. in Raw Vegetables: Molecular and Phenotypical Analysis of Their Antimicrobial Resistance and Virulence-Related Traits
Source: Int J Mol Sci. 2021 Nov 23;22(23):12626. doi: 10.3390/ijms222312626 (PMC8657893; doi:10.3390/ijms222312626)
Supplement: Supplementary file 1 [file ijms-22-12626-s001.zip › ijms-1440592-supplementary.pdf]

Occurrence of *Pseudomonas* spp. in raw vegetables: molecular and phenotypical analysis of their antimicrobial resistance and virulence-related traits

Lidia Ruiz-Roldán[1,a]; Beatriz Rojo-Bezares[1]; Carmen Lozano[1,b]; María López[1]; Gabriela Chichón[1]; Carmen Torres[2]; Yolanda Sáenz[1*]

[1] Centro de Investigación Biomédica de La Rioja (CIBIR), C/ Piqueras 98, 26006, Logroño, Spain

[2] Área de Bioquímica y Biología Molecular, Universidad de La Rioja, C/Madre de Dios, 51, 26006, Logroño, Spain

[a] Present address: Joint Research Unit "Infection and Public Health" FISABIO-University of Valencia, Institute for Integrative Systems Biology I2SysBio (CSIC-UV), Av. de Catalunya, 21, 46020 Valencia, Spain

[b] Present address: Área de Bioquímica y Biología Molecular, Universidad de La Rioja, C/ Madre de Dios 51, 26006, Logroño, Spain

Table S1. Positive samples for *Pseudomonas* spp. isolates, including their antimicrobial resistance phenotype and Pulsed-Field Gel Electrophoresis (PFGE) patterns.

Origin	Sample Name	Species (Nº of isolates)	Strain	Resistance phenotype[a]	PFGE Pattern
Lettuce	AV2	*P. mendocina*	**Ps714**	ATM	P38
		P. putida	**Ps715**	ATM	P64
		P. mendocina (2)	**Ps716**	Susceptible	P37
		P. mendocina	**Ps731**	Susceptible	P41
	AV16	*P. mendocina*	**Ps725**	Susceptible	P40
		P. aeruginosa (2)	**Ps733**	Susceptible[†]	P1
	AV20	*P. aeruginosa*	**Ps734**	Susceptible[†]	P12
		P. alcaligenes	**Ps735**	DOR	P114
		P. mendocina	**Ps736**	Susceptible	P42
	AV21	*P. otitidis*	**Ps737**	DOR	P115
		P. mendocina	**Ps738**	Susceptible	P43
	AV32	*P. putida*	**Ps729**	ATM	P67
	AV39	*P. plecoglossicida*	**Ps730**	ATM	P113
		P. mendocina	**Ps746**	ATM	P44
	AV46	*P. mendocina*	**Ps748**	Susceptible	P45
		P. mendocina	**Ps749**	Susceptible	P46
	AV56	*P. plecoglossicida*	**Ps755**	ATM	P119
	AV65	*P. aeruginosa*	**Ps760**	Susceptible[†]	P24
		P. mendocina	**Ps761**	ATM	P47
	AV75	*P. plecoglossicida*	**Ps770**	ATM	P122
	AV76	*P. mendocina*	**Ps771**	Susceptible	P49
		P. putida	**Ps772**	ATM	P79
		P. putida	**Ps773**	Susceptible	P80
		P. aeruginosa (2)	**Ps775**	Susceptible[†]	P4
		P. mendocina	**Ps776**	Susceptible	P63
	AV86	*P. mendocina*	**Ps782**	Susceptible	P50
		P. plecoglossicida	**Ps783**	ATM	P125
	AV90	*P. aeruginosa*	**Ps786**	Susceptible[†]	P26
	AV97	*P. aeruginosa*	**Ps794**	Susceptible[†]	P8
		P. aeruginosa	**Ps795**	Susceptible[†]	P9
	AV105	*P. mendocina* (2)	**Ps799**	Susceptible	P93
	AV111	*P. aeruginosa* (2)	**Ps805**	Susceptible[†]	P10
		P. mendocina	**Ps806**	Susceptible	P55
		P. mendocina	**Ps807**	Susceptible	P56
	AV117	*P. putida*	**Ps877**	ATM	P94
	AV130	*P. plecoglossicida*	**Ps900**	ATM	P133

Origin	Sample Name	Species (Nº of isolates)	Strain	Resistance phenotype[a]	PFGE Pattern
Cucumber	AV8	*P. aeruginosa*	**Ps720**	Susceptible[†]	P11
	AV14	*P. alcaliphila*	**Ps916**	Susceptible	P137
	AV18	*P. plecoglossicida*	**Ps727**	ATM	P112
	AV34	*P. plecoglossicida*	**Ps742**	ATM	P117
		P. putida	**Ps743**	ATM	P70
	AV49	*P. putida* (2)	**Ps753**	ATM	P73
	AV71	*P. plecoglossicida*	**Ps763**	ATM	P120
	AV93	*P. monteilii*	**Ps789**	ATM	P126
	AV110	*P. aeruginosa*	**Ps804**	Susceptible[†]	P30
		P. putida	**Ps843**	Susceptible	P91
	AV112	*P. putida*	**Ps873**	PIP, ATM	P92
		P. putida	**Ps874**	ATM	P92
		P. putida	**Ps895**	MEM	P92
		P. plecoglossicida	**Ps896**	ATM	P132
Zucchini	AV11	*P. putida*	**Ps723**	Susceptible	P66
	AV29	*P. putida*	**Ps740**	ATM	P69
	AV70	*P. putida*	**Ps762**	ATM	P76
	AV94	*P. putida*	**Ps790**	ATM	P86
	AV103	*P. putida*	**Ps841**	Susceptible	P88
		P. monteilii	**Ps871**	Susceptible	P129
		P. putida	**Ps872**	ATM	P89
	AV116	*P. fluorescens*	**Ps876**	CAZ, IPM, MEM, ATM, DOR[†]	P130
	AV133	*P. aeruginosa* (2)	**Ps848**	Susceptible[†]	P17
		P. aeruginosa (2)	**Ps883**	Susceptible[†]	P32
	AV139	*P. putida*	**Ps885**	Susceptible	P99
		P. monteilii	**Ps910**	ATM	P136
		P. aeruginosa (2)	**Ps854**	Susceptible[†]	P20
		P. aeruginosa	**Ps855**	Susceptible[†]	P21
Onion/Leek	AV3	*P. monteilii*	**Ps718**	ATM	P109
	AV4	*P. plecoglossicida*	**Ps719**	ATM	P110
	AV36	*P. putida*	**Ps744**	Susceptible	P71
	AV62	*P. putida*	**Ps758**	ATM	P74
	AV72	*P. monteilii*	**Ps765**	ATM	P121
		P. aeruginosa	**Ps764**	Susceptible[†]	P25
	AV81	*P. putida*	**Ps780**	ATM	P83
	AV120	*P. putida*	**Ps898**	ATM	P104
Potato	AV17	*P. plecoglossicida*	**Ps726**	ATM	P111
	AV37	*P. putida*	**Ps745**	ATM	P72
	AV61	*P. aeruginosa*	**Ps756**	Susceptible[†]	P3

Origin	Sample Name	Species (Nº of isolates)	Strain	Resistance phenotype[a]	PFGE Pattern
		P. aeruginosa	**Ps757**	Susceptible[†]	P5
	AV63	*P. putida*	**Ps759**	Susceptible	P75
	AV80	*P. plecoglossicida*	**Ps777**	ATM	P123
		P. putida	**Ps778**	ATM	P81
		P. putida	**Ps779**	Susceptible	P82
	AV85	*P. plecoglossicida*	**Ps781**	ATM	P124
	AV89	*P. mendocina*	**Ps784**	Susceptible	P51
		P. putida	**Ps785**	Susceptible	P84
	AV96	*P. aeruginosa*	**Ps791**	Susceptible[†]	P6
		P. aeruginosa (2)	**Ps793**	Susceptible[†]	P7
	AV100	*P. putida*	**Ps797**	Susceptible	P87
	AV109	*P. chlororaphis*	**Ps917**	Susceptible	P138
	AV118	*P. putida* (2)	**Ps879**	ATM	P95
		P. putida	**Ps897**	ATM	P96
Green bean	AV12	*P. putida*	**Ps732**	ATM	P68
	AV28	*P. oryzihabitans*	**Ps739**	Susceptible	P116
	AV73	*P. putida*	**Ps766**	ATM	P77
	AV98	*P. aeruginosa*	**Ps796**	MEM. ATM[†]	P27
		P. punonensis	**Ps840**	Susceptible	P128
	AV104	*P. aeruginosa*	**Ps798**	Susceptible[†]	P28
	AV106	*P. aeruginosa*	**Ps801**	Susceptible[†]	P29
		P. putida	**Ps842**	ATM	P90
	AV138	*P. aeruginosa* (2)	**Ps851**	Susceptible[†]	P18
		P. aeruginosa	**Ps852**	Susceptible[†]	P19
		P. aeruginosa	**Ps884**	IPM[†]	P33
	AV141	*P. plecoglossicida*	**Ps886**	ATM	P131
	AV143	*P. aeruginosa*	**Ps837**	Susceptible[†]	P15
		P. putida (2)	**Ps911**	ATM	P100
	AV145	*P. putida*	**Ps891**	ATM	P102
Chard	AV10	*P. mendocina*	**Ps721**	ATM	P39
		P. putida	**Ps722**	Susceptible	P65
	AV31	*P. mendocina*	**Ps728**	Susceptible	P108
		P. mendocina	**Ps741**	ATM	P53
	AV40	*P. plecoglossicida*	**Ps747**	ATM	P118
	AV47	*P. aeruginosa* (3)	**Ps752**	Susceptible[†]	P2
	AV74	*P. mendocina* (2)	**Ps767**	Susceptible[†]	P48
		P. putida	**Ps768**	ATM	P78
	AV92	*P. mendocina*	**Ps787**	Susceptible	P52
		P. putida	**Ps788**	ATM	P85

Origin	Sample Name	Species (Nº of isolates)	Strain	Resistance phenotype[a]	PFGE Pattern
	AV108	*P. mendocina*	**Ps802**	Susceptible	P54
		P. plecoglossicida	**Ps803**	ATM	P127
	AV115	*P. putida*	**Ps875**	ATM	P139
	AV122	*P. aeruginosa* (2)	**Ps845**	Susceptible†	P16
		P. putida	**Ps899**	ATM	P105
	AV132	*P. aeruginosa*	**Ps846**	Susceptible†	P22*
		P. putida	**Ps880**	ATM	P97
		P. putida	**Ps881**	ATM	P98
		P. monteilii	**Ps901**	ATM	P134
		P. mendocina	**Ps902**	ATM	P57
		P. putida	**Ps903**	ATM	P106
	AV137	*P. aeruginosa*	**Ps849**	Susceptible†	P22*
		P. mendocina	**Ps904**	Susceptible	P58
		P. mendocina	**Ps905**	Susceptible	P59
		P. mendocina	**Ps906**	Susceptible	P60
		P. plecoglossicida	**Ps907**	ATM	P135
		P. mendocina	**Ps908**	Susceptible	P61
		P. mendocina	**Ps909**	Susceptible	P62
	AV144	*P. aeruginosa*	**Ps857**	Susceptible†	P23
		P. putida (3)	**Ps890**	ATM	P101
	AV146	*P. aeruginosa* (2)	**Ps838**	Susceptible†	P13
		P. aeruginosa	**Ps839**	IPM†	P14
		P. aeruginosa	**Ps892**	Susceptible†	P34
		P. putida	**Ps912**	ATM	P107
	AV147	*P. aeruginosa*	**Ps858**	Susceptible†	P31
		P. aeruginosa	**Ps893**	Susceptible†	P35
		P. aeruginosa	**Ps913**	Susceptible†	P36
		P. putida	**Ps894**	ATM	P103

[a]Susceptible: This strain was susceptible to all antibiotics tested; CAZ, ceftazidime; ATM, aztreonam; IPM, imipenem; MEM, meropenem; DOR, doripenem; PIP, piperacillin.
† Presence of inducible AmpC phenotype
* Ps846 and Ps849 showed the same PFGE pattern but they were recovered from different chard samples.

Table S2. Polymorphisms detected in porin OprD of the 37 *P. aeruginosa* strains isolated from food vegetables. *P. aeruginosa* PAO1 (GenBank accession number AE004091) was used as reference strain.

Strains (Nº total)	OprD Size	OprD Pattern[a]	Amino acid Changes
Ps851; Ps913 (2)	443	A	WT
Ps734; Ps752; Ps756; Ps757; Ps764; Ps775; Ps786; Ps791; Ps794; Ps795; Ps798; Ps801; Ps804; Ps805; Ps837; Ps838; Ps839; Ps845; Ps854; Ps857; Ps858; Ps892; Ps893 (23)	441	B	D43N; S57E; S59R; E202Q; I210A; E230K; S240T; N262T; A267S; A281G; K296Q; Q301E; R310G; V359L; Loop L7-short
Ps796; Ps855; Ps883 (3)	443	C	T103S; K115T; F170L; E185Q; P186G; V189T; R310E; A315G; G425A
Ps733 (1)	443	D	T103S; K115T; V127L; F170L; E185Q; P186G; V189T; R310E; A315G; G425A
Ps793 (1)	441	E	V127L; E185Q; P186G; V189T; E202Q; I210A; E230K; S240T; N262T; T276A; A281G; K296Q; Q301E; R310E; G312R; A315G; L347M; Loop L7-short; S403A; Q424E
Ps720 (1)	443	F	T103S; K115T; F170L
Ps848 (1)	443	G	D43N; T103S; K115T; F170L
Ps760 (1)	443	H	V127L; F170L
Ps846; Ps849 (2)	443	I	D43N
Ps852 (1)	443	J	A293T
Ps884 (1)		K	IS*Pa1635* at nucleotide 561

[a]WT: wild type, strains described the same amino acid pattern as *P. aeruginosa* PAO1 reference strain.

Table S3. Virulence patterns detected among the 37 *P. aeruginosa* strains isolated from different food vegetables.

Nº of strains	Amplification of genes															Virulence pattern
	exoU	*exoS*	*exoY*	*exoT*	*exlA*	*exoA*	*lasA*	*lasB*	*aprA*	*rhlAB*	*rhlC*	*rhlI*	*rhlR*	*lasI*	*lasR*	
23	-	+	+	+	-	+	+	+	+	+	+	+	+	+	+	I
1	-	+	+	+	-	+	+	+	+	+	+	+	+	+	+[a]	IIa
1	-	+	+	+	-	+	+	+	+	+	+	+	+	+	+[b]	IIb
1	-	+	+	+	-	+	+	+	+	+	+	+	+	+	+[c]	IIc
2	-	+	-	+	-	+	+	+	+	+	+	+	+	+	+	III
3	-	+	+	+	-	+	+	+	+	+	+	+	+	-	-	IV
2	+	-	+	+	-	+	+	+	+	+	+	+	+	+	+	V
1	+	-	-	+	-	+	+	+	+	+	+	+	+	+	+[d]	VI
2	-	-	+	+	-	+	+	+	+	+	+	+	+	+	+	VII
1	+	-	+	+	-	-	+	+	+	+	+	+	+	+	+	VIII

[a] *lasR* gene truncated by IS*1411* element.
[b] *lasR* gene truncated by IS*Pst7* element.
[c] *lasR* gene of 647 bp instead of 720 bp.
[d] *lasR* gene truncated by IS*Pre2* element.

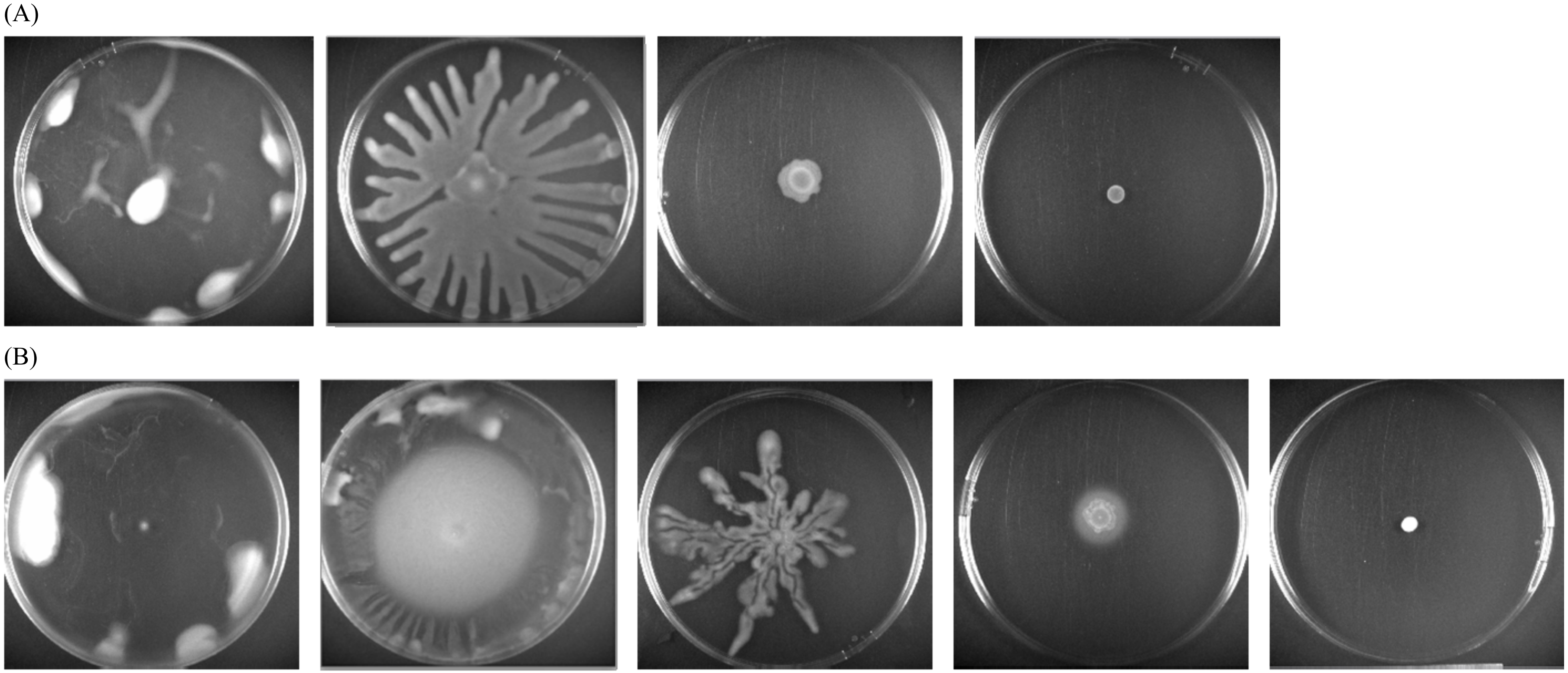

Figure S1. Different swarming (A) and swimming (B) motility patterns detected in the 37 *P. aeruginosa* strains.

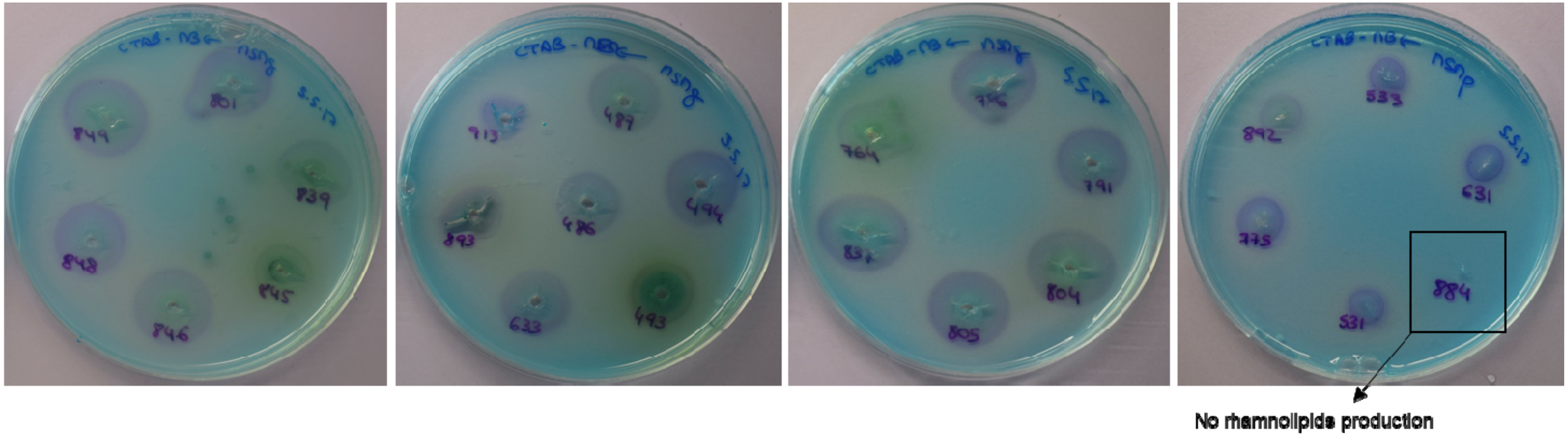

Figure S2. Rhamnolipids production in *P. aeruginosa* strains. The experiment was carried out in Cetyl Trimethylammonium Bromide – Methylene Blue (CTAB-MB) agar plates. Ps884 strain (square) did not show rhamnolipids production.
